# Study on Shear Performance of Corroded Steel Fiber Reinforced Concrete Beams under Impact Load

**DOI:** 10.3390/ma17112566

**Published:** 2024-05-27

**Authors:** Jianxiao Gu, Liancheng Li, Xin Huang, Hui Chen

**Affiliations:** 1 Wenzhou Key Laboratory of Intelligent Lifeline Protection and Emergency Technology for Resilient City, College of Architecture and Energy Engineering, Wenzhou University of Technology, Wenzhou 325035, China; 102015295@glut.edu.cn; 2 College of Architecture and Civil Engineering, Wenzhou University, Wenzhou 325035, China; 21461544036@stu.wzu.edu.cn; 3 Wenzhou Traffic Engineering Testing and Testing Co., Ltd., Wenzhou 325035, China; 20461542031@stu.wzu.edu.cn

**Keywords:** impact performance, SFRC beams, failure mode, dynamic response, energy dissipation

## Abstract

With the growing use of steel-fiber-reinforced-concrete (SFRC) beams in environmentally friendly and rapid construction, it is essential to assess their impact performance. These beams may encounter unexpected impact loadings from accidents or terrorist attacks during service life. This study explored the impact of steel fiber content and drop hammer height on the impact load testing of corrosion-treated SFRC beams. Experiments were conducted with varying steel fiber contents (0%, 0.25%, 0.5%, 0.75%, and 1.0%), and drop hammer height (1 m, 2 m, and 3 m). The corrosion test demonstrates that SFRC beams supplemented with steel fibers showcase a diminished surface rust spot area in comparison to those lacking fibers. This improvement is ascribed to the bonding between fibers and the concrete matrix, along with their current-sharing properties. SFRC beams, subjected to impact testing, exhibit concrete crushing at the top without spalling, showcasing improved impact resistance due to increased fiber content, which reduces crack formation. Additionally, different fiber contents yield varied responses to impact loads, with higher fiber content notably enhancing overall beam performance and energy dissipation capacity. Energy dissipation analysis shows a moderate increase with higher fiber contents, and impulse impact force generally rises with fiber content, indicating improved impact resistance.

## 1. Introduction

Worldwide, the extensive utilization of reinforced concrete (RC) structures can be primarily attributed to the favorable properties of concrete. These encompass high ductility, considerable stiffness, minimal maintenance requirements, enhanced fire resistance, and significant compressive strength despite its inherent deficiency in tensile strength [1,2,3]. However, corrosion poses a significant threat to the durability of RC structures, impacting both mechanical strength and long-term performance [4]. Normally, rust formation weakens the bond between steel and concrete, causing delamination and spalling. Moreover, corrosion-induced volumetric expansion imposes splitting stresses on surrounding concrete, resulting in cracking and eventual cover spalling. Increased exposure exacerbates corrosion rates, accelerating structural deterioration. To mitigate the impact of corrosion on durability and ensure the structure’s long-term service life, significant investments are necessary for maintenance and repair [5].

In recent years, the world has experienced an increase in accidents, earthquakes, heavy objects, or rockfalls impacting on concrete slabs, as well as terrorist attacks, landslides, floods, and incidents involving the release of corrosive gases and liquids, all of which have caused damage to concrete structures [6,7,8,9,10]. Moreover, rust and corrosion phenomena typically manifest in moist environments with the presence of oxygen, along with salts in the soil or groundwater [11,12]. Understanding the response of corroded reinforced concrete (RC) beams to impact loads is essential in assessing their ability to withstand unexpected forces, such as those encountered during accidents or deliberate acts. In light of the escalating frequency of unpredictable and extreme events, enhancing the resilience and safety of structures necessitates thorough research into the behavior of concrete beams under impact conditions [13,14,15,16,17,18]. The above-mentioned research has paid little attention to the mechanical behavior of concrete beams under impact loads in the presence of rebar corrosion [19]. The weakening of the bond between steel and concrete due to rust formation leads to delamination and spalling. Therefore, the objective of this study is to examine the mechanical response of RC beams affected by corrosion when subjected to impact loading.

Recognizing the potential severe issues arising from the corrosion of stirrups, numerous studies have been conducted to examine the performance of RC beams containing corroded stirrups [2,20,21,22,23,24,25,26]. The presence of corroded stirrups compromises the shear capacity and ductility of RC beams, with the shear span ratio, stirrup spacing, and concrete strength being significant influencing factors. Research conducted by Fernandez et al. [27] and Al-Sibahy et al. [28] using electrically accelerated corrosion has revealed a correlation between the reduction in load-carrying capacity and the corrosion-induced loss of cross-sectional areas in steel reinforcement. Moreover, the corrosion-induced structural deterioration and defects in steel reinforcement have led to a significant need for maintenance in numerous concrete highway bridges. Extending the service life of corroded RC structures involves repairing them to enhance both their load-bearing capacity and deformation resistance [23,26]. Several strengthening technologies, including fiber-reinforced polymer (FRP), steel plates, and minor concrete repair, are available for restoring degraded RC structures. For instance, experiments by Chen et al. [29] on RC specimens immersed for 60 days in a 10% Na_2_SO_4_ solution showed a loss of load-bearing capacity of 17.8%, which rose to 34.8% under coupled cyclic loading. Lu et al. [30] also observed alterations in crack pattern, ductility, and stiffness evolution in beams experiencing corrosion-induced mass losses ranging from 4% to 18%. Nevertheless, there has been relatively limited investigation into the impact loading behavior resulting from dynamic loads in corroded RC beams [20,31,32]. This study conducted impact load tests, considering the variables of steel fiber content, corrosion conditions, and impact load level, to investigate the mechanical response and analyze the test results. 

Moreover, extensive research has explored detailed experimental, numerical, and analytical methods to investigate the dynamic response of fiber-reinforced-concrete (FRC) beams [1,16,33,34,35,36,37,38]. For instance, considerable attention has been devoted to investigating the performance of concrete beams reinforced with Glass FRP (GFRP) bars, particularly under impact loading conditions [39,40,41]. The results indicated that these beams exhibited an average 15% increase in moment capacity under impact loading (using a drop mass of 110 kg at a height of 1.2 m) compared to static loading conditions. While this research has shed light on the mechanical response of FRC beams while considering various variables, it has not been comprehensive in its approach. However, there has been limited research in the last decade concerning the impact of drop hammer height, corrosion condition, and fiber content on concrete structures. Examining these factors is vital for assessing concrete structural safety. This study investigates the mechanical response and strength parameters of corroded steel-fiber-reinforced-concrete (SFRC) beams under different impact energy and fiber content in an impact load test.

This study explores the mechanical behavior of SFRC beams, examining the influence of corrosion condition, steel fibers content, and impact energy on their properties through drop hammer impact load tests. The study includes fifteen specimens for drop hammer impact tests. It monitors and compares failure modes, midspan deflection, impact force, and absorbed energy. Additionally, impact mechanical behavior responses based on test data are analyzed to assess the beam’s damage level under loadings. The failure modes of SFRC beams are investigated using high-speed camera images, and energy dissipation is calculated. Overall, the inclusion of steel fibers in SFRC beams improves impact resistance and deformation capacity, enhancing structural performance under impact loading conditions. Conclusions drawn from this paper will enhance understanding of the differential impact response of SFRC beams under varying steel fiber contents and impact energies.

## 2. Experimental Program

### 2.1. Materials

**Figure 1:**
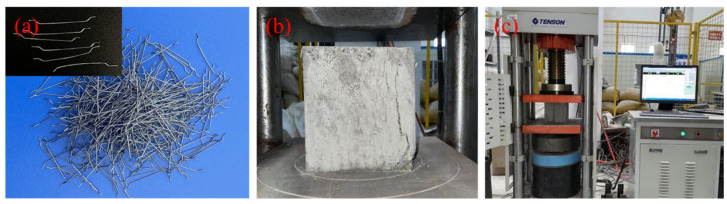
Materials and steel-fiber-reinforced concrete cube specimens, (**a**) steel fiber (SF) material, (**b**) steel-fiber-reinforced-concrete cube specimens, and (**c**) compressive strength tests device diagram.

The experiment employed ordinary Portland cement, and the results of compressive strength tests for concrete cube specimens are detailed in Table 1 (following the testing method outlined in GB/T 50081-2002 [42]). This in turn gives a concrete mixture with a mix proportion of 1:1.5:2.8:4.7 (water–cement–sand–gravel) by weight. The composition of each part is controlled proportionally based on the total mass of the RC beams. Natural river sand was used as the fine aggregate, while the coarse aggregate was crushed limestone with a maximum size of 20 mm. The study utilized high-performance cold-drawn hooked end steel-fibers with a length of 30 mm, a diameter of 0.5 mm, a density of 7800 kg/m^3^, and an ultimate tensile strength of 1200 MPa. Five dosages of steel fibers were adopted, at 0%, 0.25%, 0.5%, 0.75% and 1% by weight of cement. The pictures of the steel fibers are presented in Figure 1, while Table 2 shows the results of compressive strength tests for steel-fiber-reinforced concrete cube specimens. For the steel bars, longitudinal steel bars (HRB400, the product comes from HBIS, Tangshan, Hebei, China) with diameters of 12 mm and 10 mm were used, displaying average yield strengths of 465 MPa and 534 MPa and ultimate yield strengths of 625 MPa and 674 MPa, respectively. Additionally, 8 mm diameter stirrup steel bars (HPB300, the product comes from HBIS, Tangshan, Hebei, China) were incorporated, exhibiting average yield strengths of 363 MPa and ultimate yield strengths of 576 MPa.

### 2.2. Details of Beam Specimens

**Figure 2:**
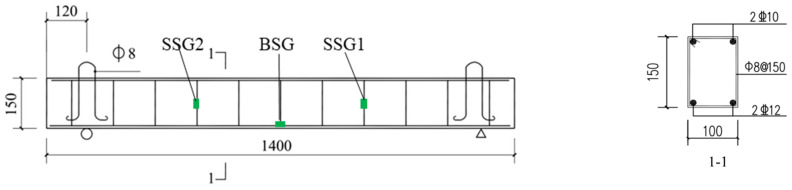
Dimensions and reinforcement configuration of SFRC beams (unit: mm).

This section presents tests conducted on a total of fifteen full-scale steel-fiber-reinforced concrete (SFRC) beams which comprise three specimens for drop hammer impact tests without steel fiber reinforcement. The dimensions of the beams, including width (b), height (h), total length (L), and clear-span length (Lc), were 100 mm, 150 mm, 1400 mm, and 1160 mm, respectively, as illustrated in Figure 2. The cover thickness for longitudinal reinforcement is 15 mm. To ease the transportation of specimens, 8 mm diameter lifting loops are installed on both sides of the SFRC beam. The stirrup reinforcement ratio (e.g., 0.670%) is designed to study the mechanical response of SFRC beams under impact loads. Additionally, one set of SFRC beams were tested with a curing period of 28 days. 

## 3. Accelerated Corrosion and Test Setup

**Figure 3:**
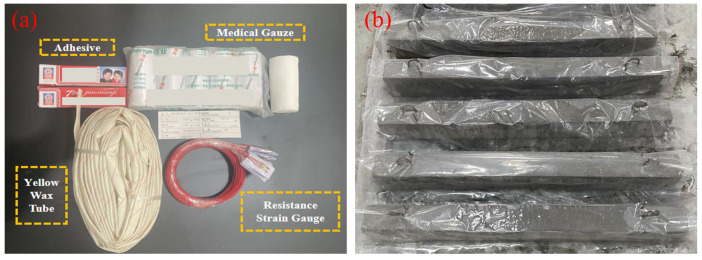
Materials and SFRC beams, (**a**) strain gauge, paste auxiliary material, (**b**) SFRC beams casting, and curing condition.

For measuring the mechanical characteristics of SFRC beams under impact loads, strain gauges (SG) are positioned at the bottom longitudinal reinforcement near the mid-span of the beam and at 1/3 span locations on the stirrup reinforcement, as illustrated in Figure 2. In the SFRC beams, three strain gauges (SGs) are affixed to the reinforcements: the bottom strain gauge (BSG) and two stirrup strain gauges (SSG1 and SSG2), as depicted in Figure 2. These gauges record strain values for analysis. Impact results, such as failure mode, peak load, load–mid-span deflection curve, and the load–strain curve of reinforcements, are subsequently examined. Two sets of strain values are measured, and their averages are calculated to enhance the reliability of the strain data. The process of installing the strain gauges involves the following steps: (1) the surface of the steel bars is polished smoothly using a grinding machine. (2) After polishing, the SGs are attached in the same direction as the steel bars using a strong adhesive, and a single-component room temperature vulcanizing silicone rubber is applied to waterproof the strain gauges. (3) Once the adhesive dries and cures, the area where the strain gauge is attached is covered with yellow waxed paper to reduce the likelihood of strain gauge failure during loading. The strain gauges (SGs) utilized are of the model BE-120-3AA (The product comes from Jiangsu Donghua Testing Technology Co., Ltd., Jingjiang, China), with a sensitivity of 2.08 ± 1%, and the materials for the SGs and attachment are depicted in Figure 3a. Additionally, after casting the specimens, they are wrapped with plastic film, and water is periodically applied after the concrete has been set to maintain humidity during the curing process. The casting and curing process of the specimens is portrayed in Figure 3b.

### 3.1. Corrosion Method

**Figure 4:**
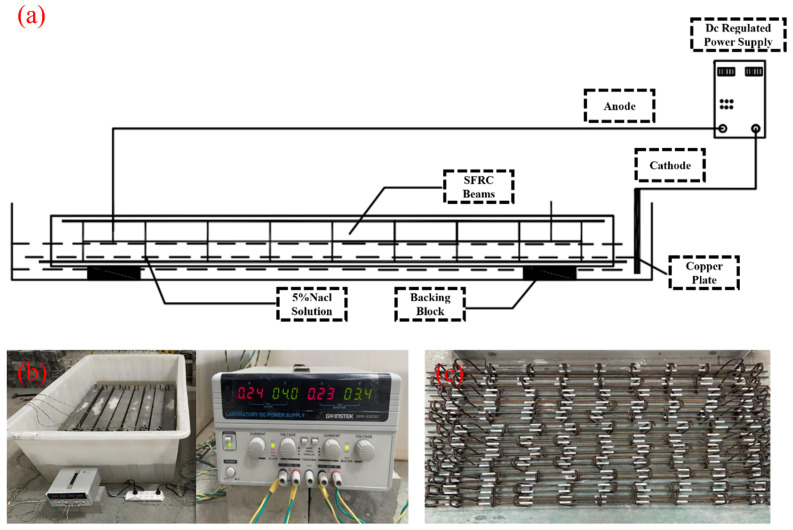
Corrosion method of SFRC beams, (**a**) test setup induced corrosion, (**b**) laboratory accelerated corrosion device, (**c**) a voltmeter device for power supply.

A composite solution containing chloride and sulfate salts was formulated to simulate the attack environment for the SFRC beams. The concentrations of these salts mirror those found in the soil and groundwater of southern Wenzhou, China. The primary constituent of the aforementioned salts was NaCl. The corrosion process was stimulated electrochemically by chloride ingress [43]. The chloride ion concentration for this class was set at 5% by mass. In accordance with the methodology outlined in this study, all SFRC beams underwent an initial curing period of 28 days. Subsequently, those SFRC beams slated for exposure to aggressive environments were subjected to a 3-day immersion in NaCl solution, as illustrated in Figure 4. This step aimed to replicate real-world conditions, allowing for a specified period before salt solutions could initiate concrete deterioration. Additionally, a copper plate was positioned beneath the SFRC beams, as depicted in Figure 4a. The corrosion process employed the copper plate as the cathode and the steel bar as the anode. Both were connected to the power supply equipment, delivering the predetermined current density as specified earlier (200 μA/cm^2^). Various SFRC beams were interconnected in a series to ensure uniform intensity, as depicted in Figure 4b. It is important to note that this study exclusively applies electrochemical acceleration corrosion to the stirrups. To facilitate this, a 4 mm diameter steel bar was employed to connect and extend the stirrups externally from the beam. The section of the steel bar in contact with the stirrups was insulated with epoxy resin and wrapped with cloth, as illustrated in Figure 4c.

The initiation of steel reinforcement corrosion was induced through the passage of an electrical current. Utilizing Faraday’s law (Equation (1)), it becomes possible to calculate the steel’s weight loss over time due to corrosion. This calculation hinges on the applied electrical current intensity, represented as I(t), along with the diameter and exposed length of the steel bar.

(1) $$E=\frac{m_{Fe}\int I\cdot dt}{V\cdot F}$$

Here, $m_{Fe}$ represents the atomic mass, V denotes the steel valency (set to two), and F stands for Faraday’s constant. Given that the applied intensity remained consistent throughout the entire test period, Faraday’s law can be restated as Equation (2) to determine the weight loss of steel.

(2) $$\Delta m=\frac{m_{Fe}I\cdot t}{V\cdot F}$$

Multiple researchers have posited that utilizing current densities below 200 μA/cm^2^ in accelerated corrosion tests yields steel weight-loss estimates comparable to those predicted by Faraday’s law, with a discrepancy of 5–10% [44,45]. The SFRC beams corrosion test in this study was conducted under electric current for 48 days. According to theoretical calculations, the corrosion rate of the stirrups was determined to be 12.3%. 

### 3.2. Impact Test

**Figure 5:**
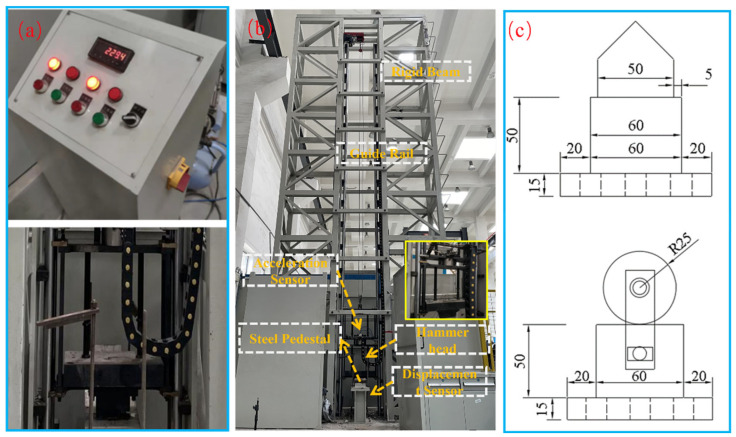
Loading device diagram, (**a**) control device, (**b**) impact load test device diagram, and (**c**) support detail (unit: mm).

**Figure 6:**
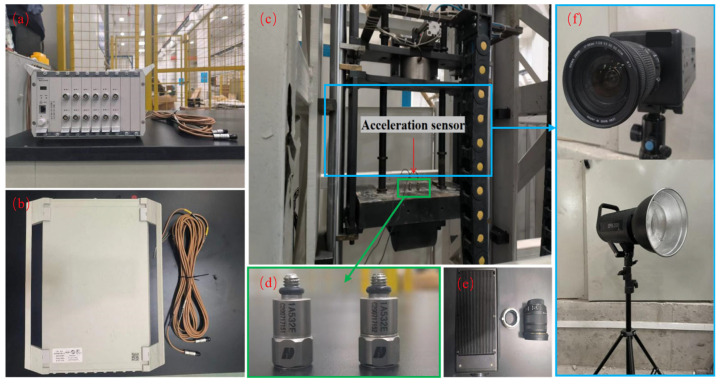
Physical diagram of the impact load test, (**a**,**b**) hyperdynamic signal test and analysis system, (**c**) drop weight impact test setup, (**d**) acceleration sensor and installation location, (**e**) high-speed camera parts, and (**f**) high-speed camera and lighting equipment.

The design and experimental procedure of the impact load test refer to GB/T 2423.5-2019 (Mechanical Shock Testing Methods and Standards) [46]. The impact tests were conducted using a drop hammer test system comprising control devices and a loading device, as depicted in Figure 5 and Figure 6. The beams, with a clear span of 1160 mm, were secured at both ends using clamps to prevent detachment from the supports during testing. A loading apparatus consisting of a cylindrical drop hammer and counterweight was utilized, with the counterweight mass being adjusted to control the impact energy. During the tests, both the hammer and counterweight were released from a predetermined height, achieving the desired speed and energy upon free-fall impact onto the beams. Additionally, the SFRC beams used in the experimental program for impact load testing are presented in Table 3. In the experiment, three identical beams were tested for each loading condition to ensure the reliability of the experiment. In the analysis, the parameters were averaged across the three beams for analysis. The beams are categorized in a DL-A-B format, wherein ‘DL’ represents the beam group, ‘A’ denotes the steel fiber content ranging from 0 to 1.0%, and ‘B’ signifies the falling height utilized in the impact load test. Data during the impact testing process are collected using the DH-5960 (The product comes from Jiangsu Donghua Testing Technology Co., Ltd., Jingjiang, China) for super-dynamic signal testing and analysis (Figure 6a,b). The DH-5960 features a total of 12 channels, each equipped with an 8 M point buffer. It boasts a maximum transient sampling rate of 20 MHz and a frequency response of up to 1 MHz. Simultaneous acquisition of acceleration, displacement, and strain data are supported. In this study, the sampling frequency is set at 100 kHz. Two channels are employed to collect acceleration data during the experiment, while the remaining five channels are dedicated to collecting displacement and strain data. The sampling frequency remains consistent across all channels.

Displacement data are gathered using the Panasonic standard-type HL-G125-A-C5 laser displacement sensor (The product comes from Jiangsu Donghua Testing Technology Co., Ltd., Jingjiang, China), featuring a measuring distance of 250 mm and a measurement range of ±150 mm. The laser displacement sensor is positioned directly beneath the center of the support steel plate. To facilitate this alignment, a circular hole is pre-drilled to enable the infrared laser emitted by the displacement sensor to pass through and align with the mid-span position of the specimen. Moreover, acceleration signals are acquired using IEPE piezoelectric accelerometer sensors with the model number 1A532E and a measurement range of ±10,000 g (The product comes from Jiangsu Donghua Testing Technology Co., Ltd., Jingjiang, China). Two identical accelerometers of the same model are simultaneously utilized during the experiment to measure the instantaneous acceleration of the hammer. This approach is adopted to reduce the risk of accelerometer failure during the test caused by vibrations induced by impacts. The acceleration sensors are depicted in Figure 6d, and their location is at the mid-span of the RC beams.

Furthermore, to document the failure characteristics of the beams during the experiment, an AE120M high-speed camera (The product comes from Jiangsu Donghua Testing Technology Co., Ltd., Jingjiang, China) is employed during the impact load testing process to capture the deformation and crack propagation of the specimens. The instrument features a full-frame resolution of 1280 × 1024 and can achieve a high frame rate of 2000 FPS. When operating in a 1024 × 128 frame, it can attain a frame rate of up to 16,000 FPS. In this experiment, the high-speed camera has a data acquisition cycle of 1000 microseconds and operates at a resolution of 1280 × 1024, as illustrated in Figure 6f.

## 4. Test Results

This section entails impact testing on SFRC beams treated for corrosion to assess the impact of stirrup ratio, drop height, and steel fiber content on shear performance. The experiments entailed gathering data on impact force, mid-span deflection, and steel strain. High-speed cameras were utilized to capture crack development in the SFRC beams, followed by analysis of corresponding time–history curves and failure modes.

### 4.1. Failure Modes

**Figure 7:**
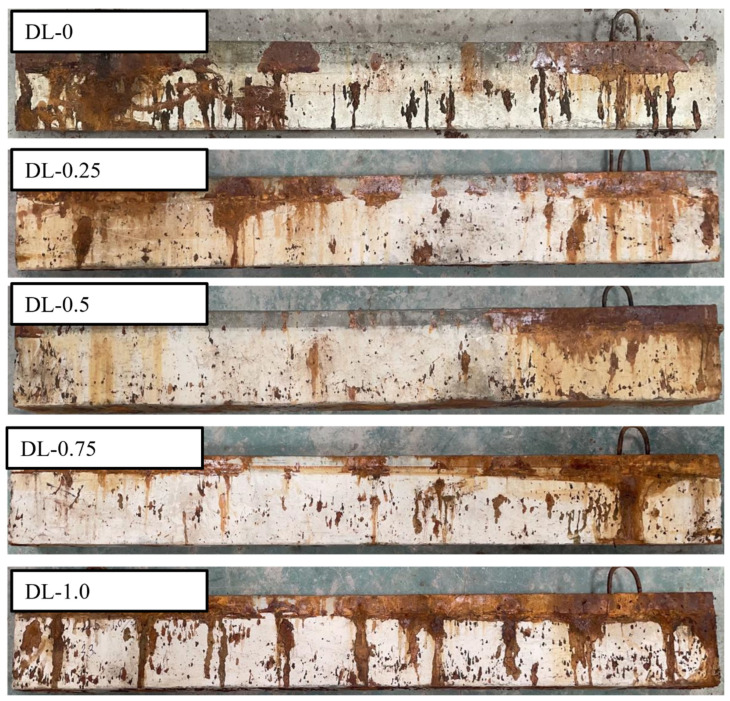
The accelerated corrosion test results of the SFRC beams. The area of rust spots on the surface of SFRC beams is less compared to the DL-0 RC beam without steel fibers.

**Figure 8:**
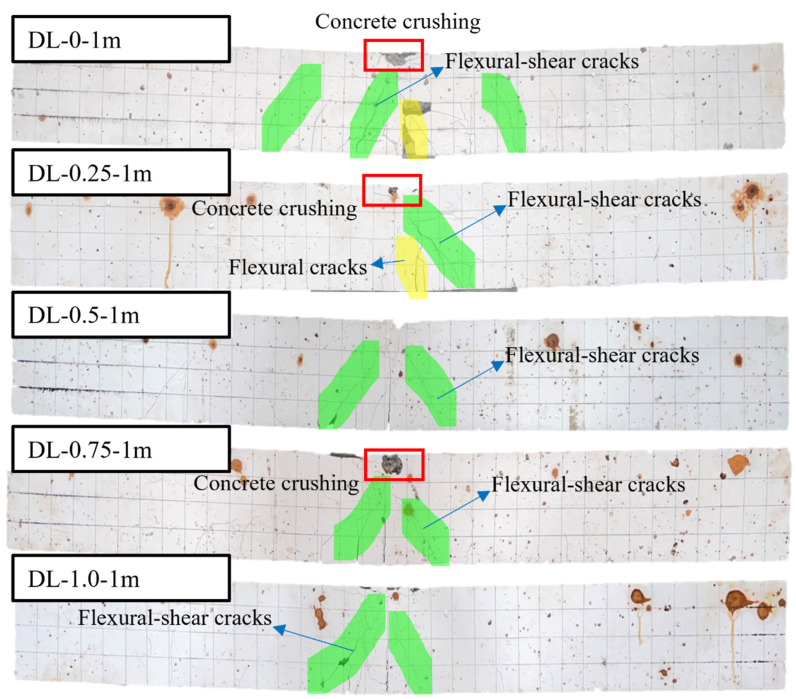
Failure modes of the corrosion-treated SFRC beams under impact loads. The failure modes of SFRC beams transition from a combined flexure failure mode to a flexural-shear mode as the steel fiber content increases from 0% to 1.0%.

**Figure 9:**
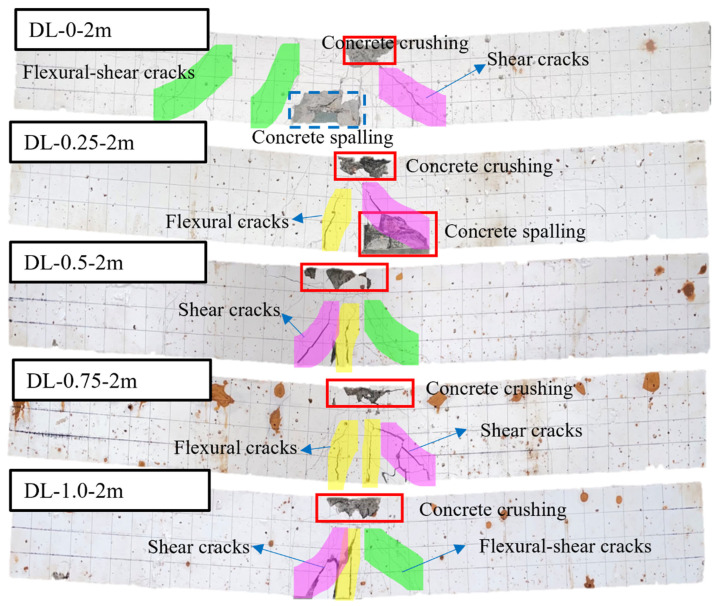
Failure modes of the corrosion-treated SFRC beams under impact loads (drop hammer height: 2 m). The SFRC beams exhibited a flexure-shear combined failure mode, characterized by broader flexural cracks at mid-span and increased post-impact residual deformation.

**Figure 10:**
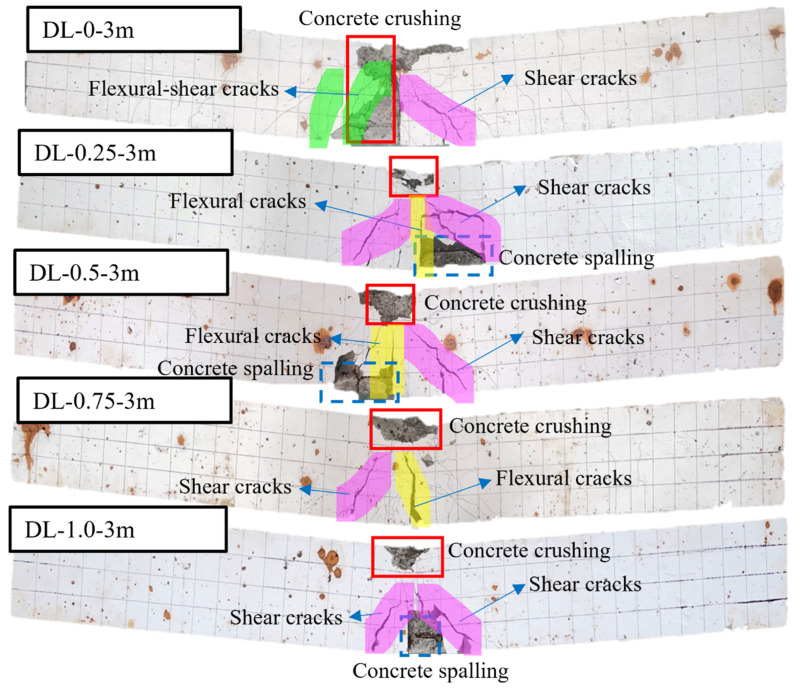
Failure modes of the corrosion-treated SFRC beams under impact loads (drop hammer height: 3 m). The failure mode transitioned from flexure-shear dominated under 2 m drop hammer height to shear dominated under impact loads, accompanied by more severe local damage, including concrete crushing on the top and spalling at the bottom.

Figure 7 illustrates the outcomes of accelerated corrosion tests conducted on SFRC beams with different steel fiber contents. The data show that SFRC beams incorporating steel fibers exhibit reduced rust spot areas on their surfaces compared to those without steel fibers (DL-0). This phenomenon is credited to the steel fibers bonding with the concrete matrix and their capacity to share the corrosion current, resulting in fewer pronounced rust marks on the concrete surface. Moreover, no expansion cracks due to rust have emerged, and the integrity of the beam specimens remains unaffected. In order to better observe the failure mode of the SFRC beams under impact load, the SFRC beams after accelerated corrosion testing were painted with white paint. The results of the impact load test are shown in Figure 8, Figure 9 and Figure 10. 

Figure 8 illustrates the failure modes of SFRC beams subjected to 1 m drop hammer impact loads with varying steel fiber contents. Concrete crushing was observed at the top of both SFRC beams, while no concrete spalling occurred at the bottom. Flexural cracks initiated at the mid-span, with subsequent development of flexure-shear cracks in the shear span region. Furthermore, the SFRC beams DL-0-1m and DL-0.25-1m experienced failure in a combined flexure-shear mode, exhibiting both flexural and flexure-shear cracks. However, an increase in steel fiber content led SFRC beams DL-0.5-1m and DL-1.0-1m to fail in a flexure-shear mode. Compared to RC beams DL-0-1m, DL-0.25-1m, DL-0.5-1m, and DL-0.5-1m, there was a reduction in the number of flexure and flexure-shear cracks. Additionally, under the 28-day curing period, most of the SFRC beams experienced diagonal shear failure, manifested by a diagonal crack on the right side of the beam extending from the point of impact to the supports. As the steel fiber content increased, the occurrence of shear cracks and flexural-shear cracks in SFRC beams during impact load tests decreased or, in some cases, vanished (as shown in Figure 8 and Figure 9).

To investigate the impact of energy levels on SFRC beams, three different impact energy levels were employed, as depicted in Figure 8, Figure 9 and Figure 10. SFRC beams DL-0-2m and DL-0-3m experienced diagonal shear failure marked by diagonal cracks on the left side of the mid-span and accompanied by concrete spalling on the bottom. SFRC beams of DL-0.25-2m, DL-0.5-2m, DL-0.75-2m, and DL-1.0-2m exhibited a flexure-shear combined failure mode, characterized by broader shear cracks at the mid-span and increased post-impact residual deformation. Furthermore, SFRC beams of DL-0.25-3m, DL-0.5-3m, DL-0.75-3m, and DL-1.0-3m exhibited a larger damaged area and more severe local damage. Specifically, SFRC beam DL-0.5-3m suffered significant splitting damage to the compression steel bars and displayed greater post-impact residual deformation at mid-span compared to SFRC Beam DL-0.5-2m, as illustrated in Figure 9 and Figure 10.

Figure 10 illustrates a transition in failure mode from flexure-shear dominance under a 2 m drop hammer height to shear dominance under higher impact loads, accompanied by more severe localized damage. The observed damage comprises concrete crushing at the top and spalling at the bottom of the SFRC beams. With higher impact loads, the beams experienced failure in diagonal shear, leading to more severe concrete crushing, additional diagonal shear cracks on both sides of the beams, and a broader distribution area of cracks. This stands in contrast to the outcomes depicted in Figure 8 and Figure 9 for SFRC beams subjected to lower impact loads. Consequently, the test results underscore the beneficial impact of higher steel fiber content on the impact performance of SFRC beams, resulting in reduced values for both maximum and residual deflection.

**Figure 11:**
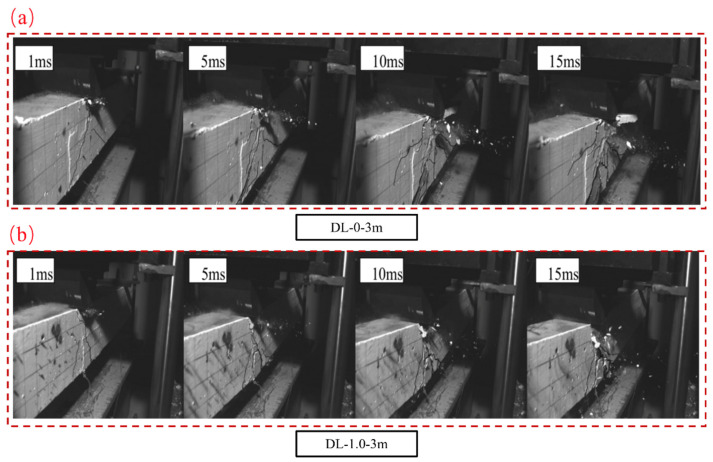
Failure process of SFRC beams recorded by a high-speed camera. The contact of the hammer on the SFRC beams is defined as 1 ms. (**a**) Failure process of SFRC beams DL-0-3m recorded. (**b**) Failure process of SFRC beams DL-1.0-3m recorded.

Due to the extensive high-speed camera data, only a subset is presented here, focusing on the DL-0-1m SFRC beam (hammer drop height: 3 m) and the DL-1.0-3m SFRC beam (hammer drop height: 3 m). Following the initial impact and the subsequent rise in mid-span bending cracks, inclined cracks penetrated the bottom of the beam. Figure 11 illustrates the failure progression of SFRC beams captured by a high-speed camera, with the contact of the hammer with the SFRC beams marked at 1 ms. Initially, during the impact load’s onset, the segment of the SFRC beam in contact with the hammer exhibited no discernible damage. For SFRC beam DL-0-3m, three flexural cracks emerged at the top of the mid-span at 1 ms. Between 5 ms and 10 ms, as mid-span flexural cracks expanded, inclined cracks propagated through the bottom of the SFRC beam, with further elongation and widening occurring. Furthermore, a fresh crack emerged on the right side of the mid-span at 10 ms. Between 10 ms and 15 ms, inclined cracks started forming from the mid-span towards the support. In the case of the DL-1.0-3m SFRC beam, one flexural crack and a lengthy longitudinal crack at the bottom were noticeable at 1 ms. Between 5 ms and 10 ms, the beam displayed a mix of flexure-shear, shear, and longitudinal cracks. By 15 ms, notably wide shear cracks appeared, leading to subsequent concrete spalling at the bottom.

Particularly noteworthy, at a 3 m hammer drop height, both SFRC Beams exhibited prominent concrete damage at 4 ms in the impact zone where the hammer contacted the beam. The pre-existing cracks gradually extended and widened, accompanied by the appearance of shorter secondary cracks. Additionally, as the impact continued, the mid-span cracks displayed a triangular distribution pattern. Furthermore, for the SFRC beam without steel fiber reinforcement subjected to impact from a 3 m drop hammer height, the extent of damage was significantly greater than that of the SFRC beam with a 1.0% steel fiber content. Taking DL-0-3m as an example, the beam exhibits pronounced diagonal cracks, with cracks intersecting to form a network-like distribution. Concrete spalling occurs at the impact location, with longitudinal reinforcement being exposed at the bottom of the beam. In contrast, for the DL-1.0-3m beam, the development of diagonal cracks is inhibited, and two main bending cracks converge to form a triangular area at the mid-span impact point where a vertical main crack is observed. There is no extensive concrete spalling in the tension zone. The mid-span deflection of the specimens without steel fibers is larger and closer to the bottom of the camera frame, indicating that the addition of steel fibers can effectively enhance the impact resistance and deformation capacity of reinforced concrete, improve its stiffness, and alter the failure mode of the concrete beam specimens, concentrating cracks at the impact location. 

### 4.2. Dynamic Responses

**Figure 12:**
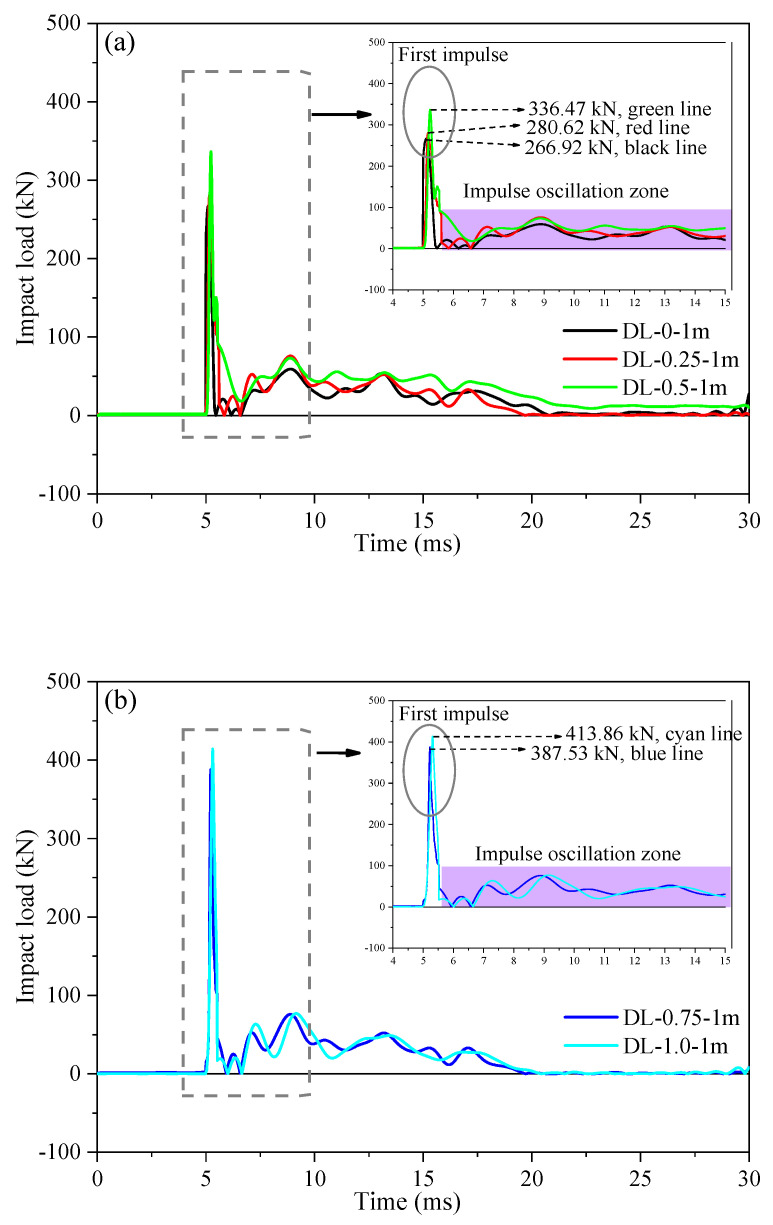

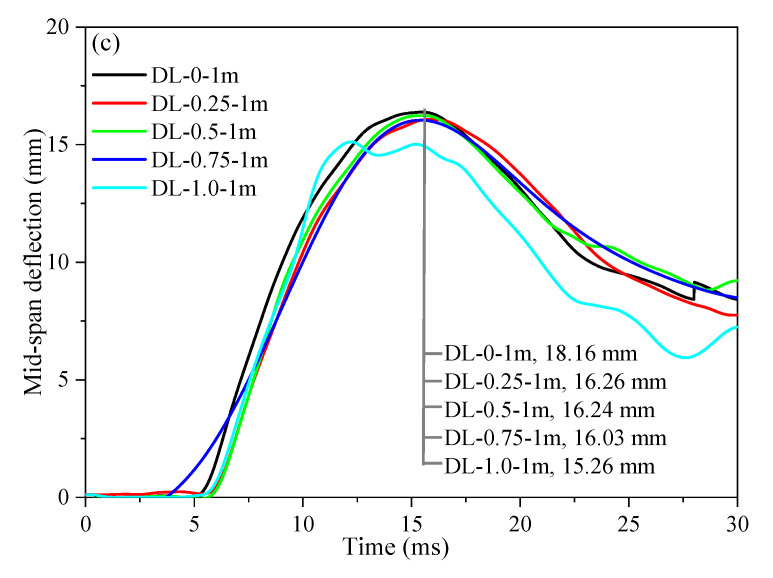
Impact force and mid-span-deflection time histories of the tested SFRC beams under the conditions of 28 d curing period and 1 m drop hammer height. (**a**) Impact force histories of SFRC beams DL-0-1m, DL-0.25-1m, and DL-0.5-1m. (**b**) Impact force histories of SFRC beams DL-0.75-1m, and DL-1.0-1m. (**c**) Mid-span-deflection time histories.

Each SFRC beam, subjected to different steel fiber contents, underwent a singular impact from the drop hammer at heights of 1 m, 2 m, and 3 m, respectively. Table 4 presents the typical impact testing results, including parameters such as impact speed (corresponding to dropping heights of 1 m, 2 m, 3 m), peak impact load, peak mid-span deflection, and residual mid-span deflection. Figure 12 displays the temporal evolution of impact loads for SFRC beams DL-0-1m, DL-0.25-1m, DL-0.5-1m, DL-0.75-1m, and DL-1.0-1m. After 48 days of accelerated corrosion under electric current, the overall stiffness of the concrete beams decreased. As depicted, the impact loads exhibit a two-impulse zone profile. Figure 12 illustrates that the SFRC beams (DL-0-1m, DL-0.25-1m, DL-0.5-1m, DL-0.75-1m, and DL-1.0-1m) underwent an initial impulse with peak values of 266.92 kN, 280.62 kN, 336.47 kN, 387.53 kN, and 413.86 kN, respectively, lasting approximately 5–6 ms. Compared to the SFRC beams of DL-0.25-1m, DL-0.5-1m, DL-0.75-1m, and DL-1.0-1m, the peak impact force increased by 5.1%, 19.9%, 15.2%, and 6.8%, respectively. A suitable amount of steel fibers can significantly enhance the overall performance of the specimen beams. Furthermore, as seen in Figure 12, this was followed by an impulse oscillation with an impact load ranging from 0 to 100 kN, lasting about 6–30 ms. This phenomenon contrasts with the findings of Huang et al. [16], who observed only two impulses in SFRC beams under impact loads. This can be explained by the splitting damage of the compression basalt-fiber-reinforced-polymer bars, which could reduce the stiffness of the beam, resulting in a lower second-peak impulse. In contrast, the present study reveals the presence of an impulse oscillation zone. Additionally, Li et al. [25] summarized the reasons in the profiles of impact loads and identified the factors influencing these profiles; however, this process was not explained in detail. Impact loading is generally characterized by the application of a force of high intensity over a brief period. Structural components under impact loading may exhibit the following two response phases: Phase one (first impulse) involves the local response to the stress wave occurring at the loading point immediately after impact. Phase two (impulse oscillation zone) involves the response, including free vibration effects due to elastic–plastic deformation occurring throughout the structural member over a prolonged period after impact. The response in the impulse oscillation zone primarily depends on the loading rate effect and the dynamic behavior of the structural component. 

**Figure 13:**
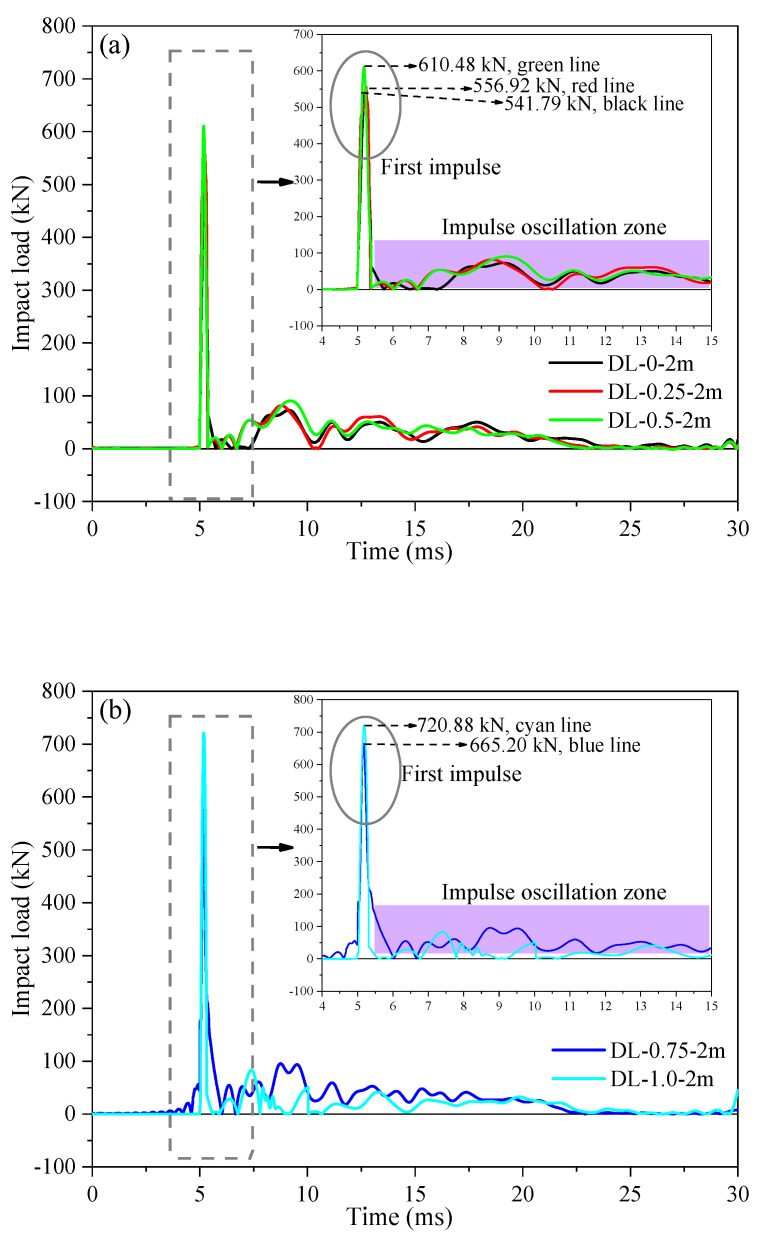

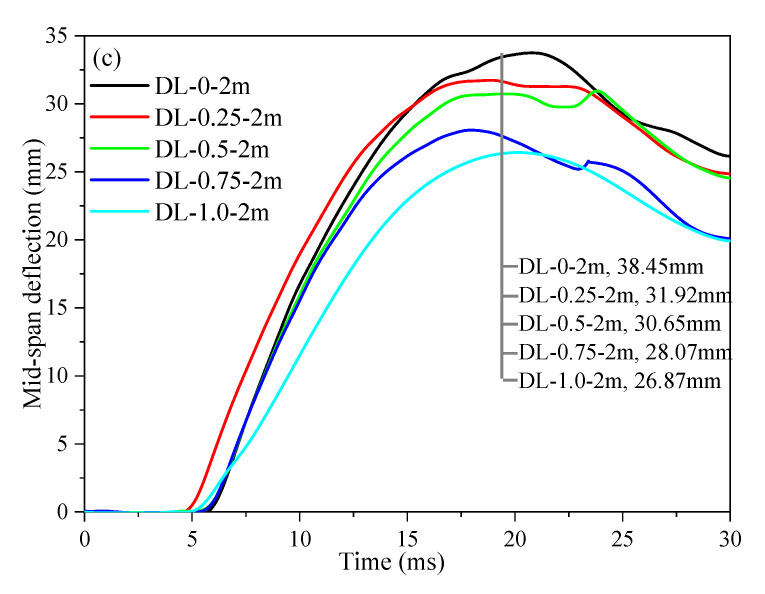
Impact force and mid-span-deflection time histories of the tested SFRC beams under the conditions of 28 d curing period and 2 m drop hammer height. (**a**) Impact force histories of SFRC beams DL-0-2m, DL-0.25-2m, and DL-0.5-2m. (**b**) Impact force histories of SFRC beams DL-0.75-2m, and DL-1.0-2m. (**c**) Mid–span–deflection time histories.

**Figure 14:**
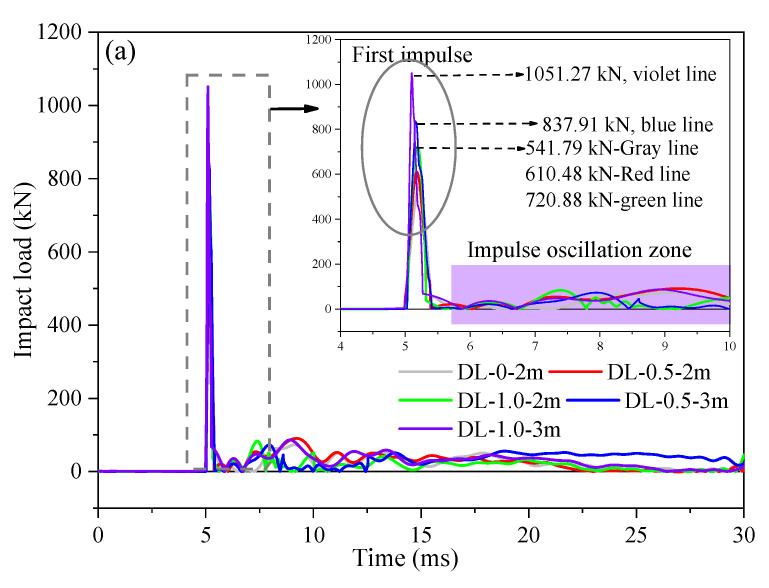

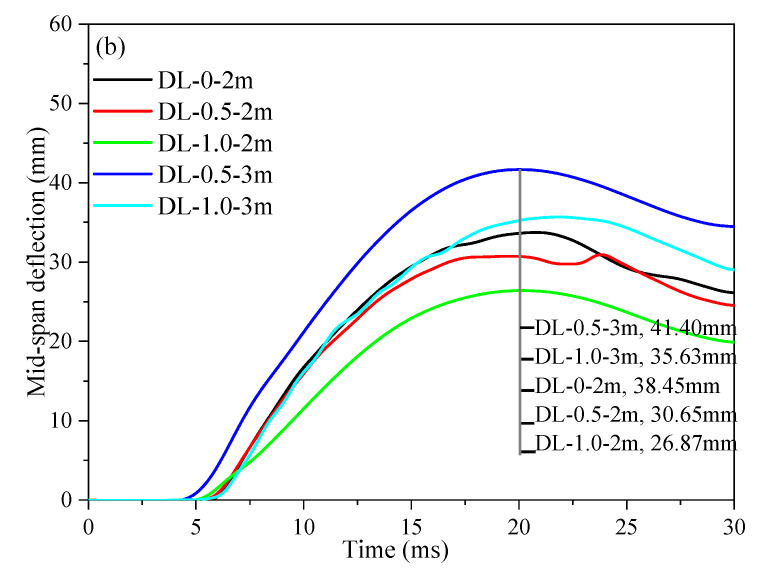
Impact force and mid-span-deflection time histories of the tested SFRC beams under the conditions of 28 d curing period, 2 m, and 3 m drop hammer height. (**a**) Impact force histories of SFRC beams. (**b)** Mid-span-deflection time histories.

Moreover, upon examination of Figure 12a,b and comparison of the peak impact load of SFRC beams, it was noted that the peak load exhibited an insignificant effect on the stiffness of SFRC beams as the steel fiber content increased during the impact test, while the peak deflection at mid-span showed a decreasing trend. This indicated that the addition of steel fibers can enhance the stiffness of the specimen beams, thereby demonstrating improved impact resistance and deformation resistance. Additionally, Figure 13 depicts how SFRC beams (DL-0-2m, DL-0.25-2m, DL-0.5-2m, DL-0.75-2m, and DL-1.0-2m) experienced an initial impulse with peak values of 514.79 kN, 556.92 kN, 610.48 kN, 665.20 kN, and 720.88 kN, respectively, lasting approximately 4–6 ms. The impulse oscillation zone, as observed in Figure 12, Figure 13 and Figure 14, is consistent, characterized by an impact load ranging from 0 to 100 kN and lasting approximately 6–30 ms. When comparing two sets of SFRC beams (one comprising DL-0.75-1m and DL-1.0-1m, and the other DL-0.75-2m and DL-1.0-2m), the response time of the peak impact load and the impulse oscillation zone ranged from approximately 4 to 6 ms and 6 to 30 ms, respectively. This variation depended on the steel fiber content and corrosion degree, influenced by the overall integrity of the SFRC beam strength and pouring process. Additionally, from Figure 14a, SFRC beams (DL-0-2m, DL-0.5-2m, DL-1.0-2m, DL-0.5-3m, and DL-1.0-3m) experienced an initial impulse with peak values of 541.79 kN, 610.48 kN, 720.88 kN, 837.91 kN, and 1051.27 kN, respectively, lasting approximately 5–6 ms. The impulse oscillation zone was more pronounced compared to the 1 m drop hammer impact test, as the hammer had to dissipate a greater amount of energy through the beam. In Figure 14, there is an increase in the peak impact load of SFRC beams with the rise in drop hammer height. For instance, the peak impact load for SFRC beam DL-0.5-3m was 837.91 kN, which exceeded that of SFRC beam DL-0.5-2m by approximately 1.37 times. Moreover, the response of the peak impact load intensified with an increase in steel fiber content. For example, the peak impact load for SFRC beam DL-1.0-3m was 1051.27 kN, which surpassed that of SFRC beam DL-0.5-3m by approximately 1.25 times.

Figure 12c, Figure 13c and Figure 14b, accompanied by Table 4, depict the mid-span deflection time histories for the tested SFRC beams. It is evident that the peak mid-span deflection decreases with an increase in steel fiber content during the impact load test, although the data for the peak mid-span deflection of the 1 m drop hammer impact test are not very clear. For instance, the peak mid-span deflections of SFRC beams DL-0-2m, DL-0.25-2m, DL-0.5-2m, DL-0.75-2m, and DL-1.0-2m are 38.45 mm, 31.92 mm, 30.65 mm, 28.07 mm, and 26.87 mm, respectively. Additionally, it is noted that the peak mid-span deflection increases with an increase in drop hammer height during the impact load test during a 28-day curing period. For example, SFRC beams DL-1.0-2m and DL-1.0-3m experience peak mid-span deflections of 26.87 mm and 35.63 mm and residual deflections of 19.83 mm and 29.58 mm, respectively. It is crucial to emphasize that the shear resistance of beams relies on various factors, including the dowel action of tension reinforcements, stirrups, interlocking aggregates, and the shear resistance of concrete in the compression zone [47]. Interestingly, SFRC beams DL-0.5-3m and DL-1.0-3m, subjected to higher drop hammer heights, exhibit larger peak and residual mid-span deflections compared to SFRC beams DL-0.5-1m and DL-1.0-1m, which experienced lower drop hammer heights. With the increase in the steel fiber content, the reduction in SFRC beam stiffness caused by the electric corrosion is mitigated. The peak impact force has been enhanced, and the increase in beam stiffness results in decreased peak deflection and residual displacement at mid-span, with rebound values fluctuating within the range of 6 to 8 mm (Figure 13c and Table 4). The addition of steel fibers to concrete can improve its impact resistance.

**Figure 15:**
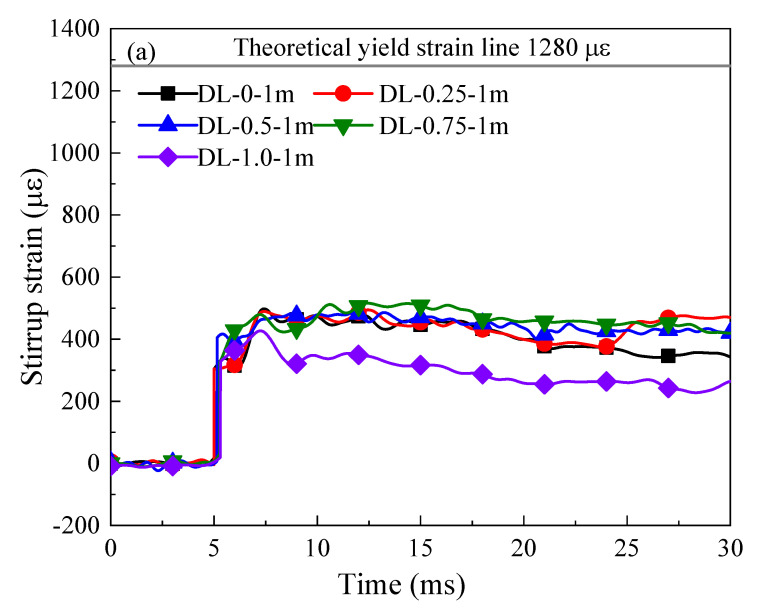

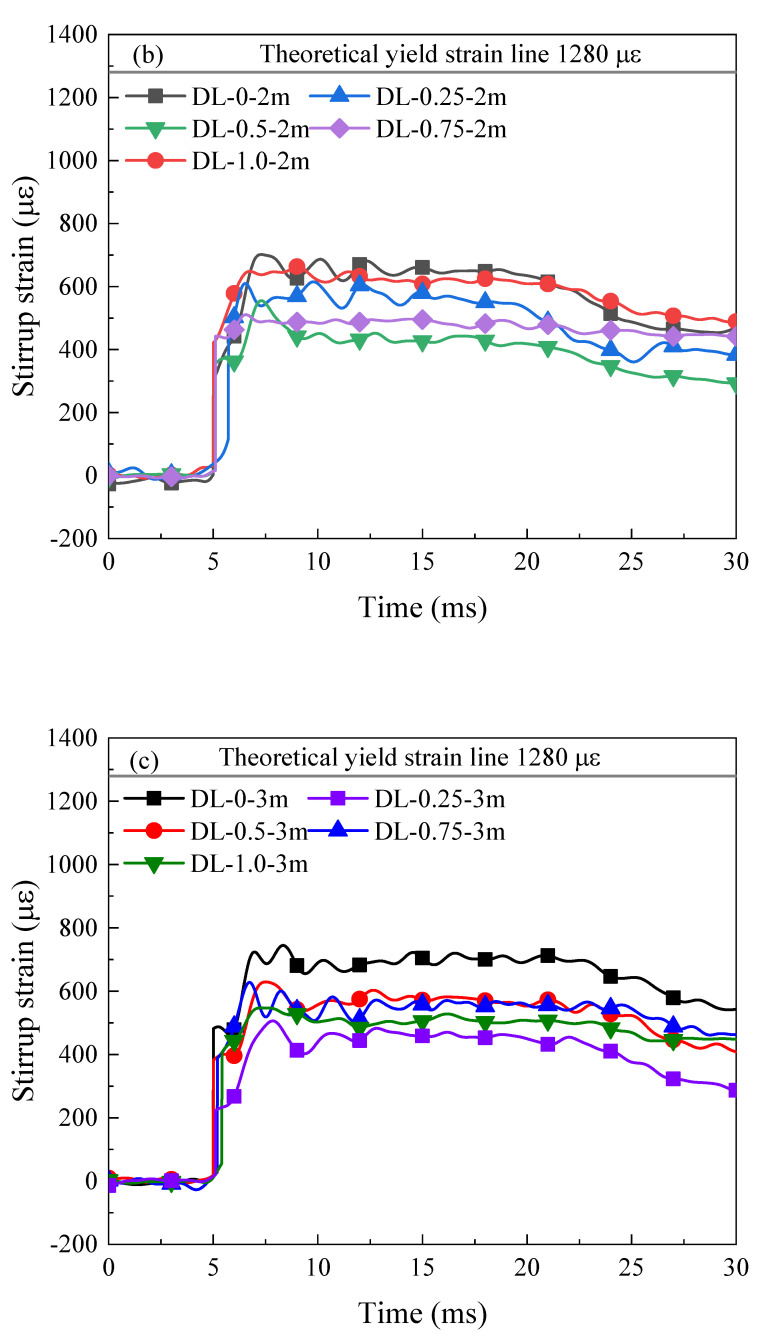
Impact load-strain curves of SFRC beams. (**a**–**c**) SFRC beams treated with varying steel fiber content reinforcement under drop hammer height 1 m, 2 m, and 3 m, respectively.

Figure 15 presents the temporal evolution of stirrup reinforcement strain for the SFRC beams subjected to impact loading. The data indicate a progressive rise in stirrup strain corresponding to the increasing drop height. Moreover, as the steel fiber content increases, the change in stirrup strain gradually diminishes, albeit with some irregularities in the data. This indicates that, under the impact load, the stress state of SFRC beams gradually shifts towards bending, resulting in a gradual decrease in the load carried by stirrups. Additionally, as shown in Figure 15, the stirrup strain at the mid-span of SFRC beams initially exhibits negative values (compressive) within the first 5 ms, attributed to stress wave propagation. Subsequently, a shift to positive values (tensile) occurs from 5 ms to 30 ms, attributed to the displacement of the neutral axis above the position of the compression steel bars. Starting from 9 ms, the concrete cover commences crushing, as illustrated in Figure 11, and is incapable of withstanding compressive stress, resulting in a downward movement of the compression zone and neutral axis. This phenomenon has been previously observed and elucidated in a study by Tran et al. [48].

**Figure 16:**
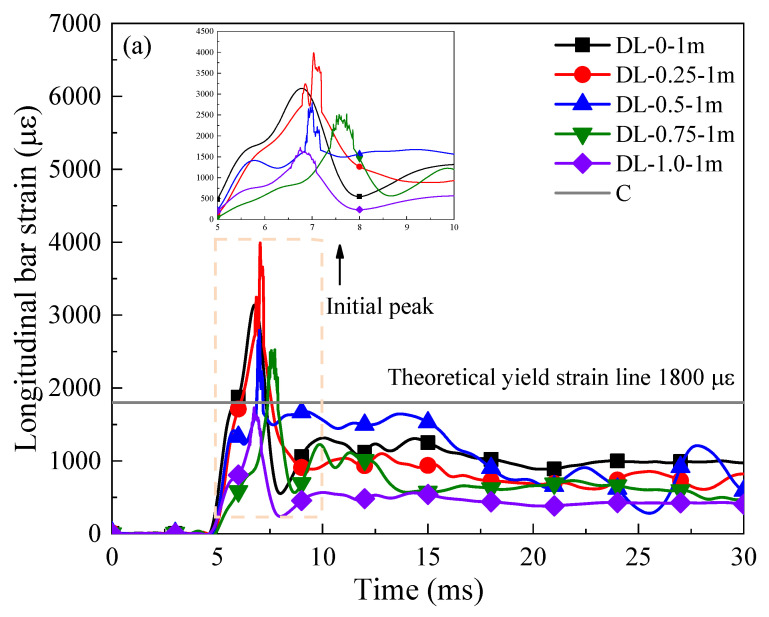

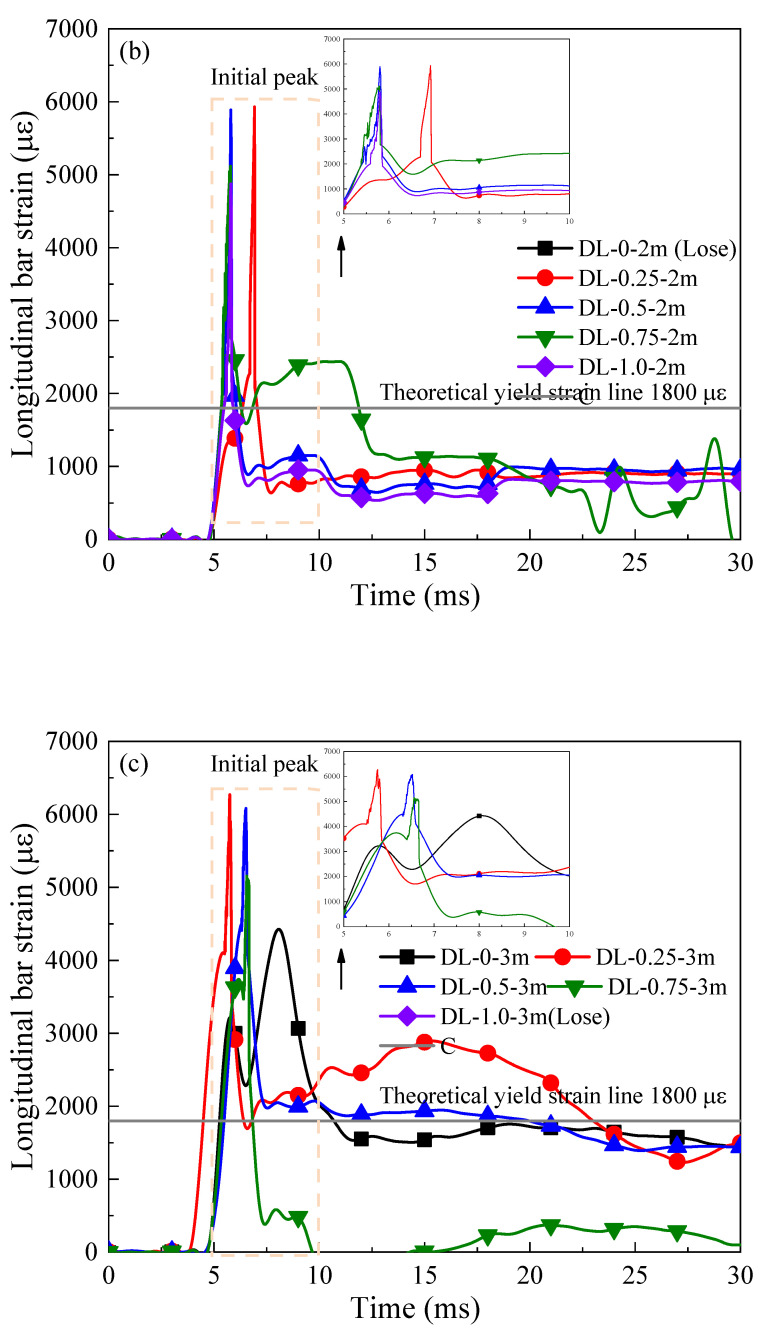
Impact load-strain curves of SFRC beams. (**a**–**c**) SFRC beams treated with varying steel fiber content reinforcement under drop hammer heights of 1 m, 2 m, and 3 m, respectively.

At the beginning of impact load (around 5 ms), the strain curve of all SFRC beams undergoes a transformation, reaching a peak value within the next 4 ms and gradually decreasing during the subsequent impact process. Throughout the entire impact process, the stirrup reinforcement remains under tension. Regarding the longitudinal strain time–history curves of the SFRC beams, let us consider the example of the longitudinal strain for SFRC beams with different steel fiber contents at dropping heights of 1 m, 2 m, and 3 m, respectively, as depicted in Figure 16. The response of longitudinal strain in the SFRC beams is slightly faster than that of stirrup strain. At 5 ms, following the impact of the drop hammerhead, the longitudinal strain begins to rise. In the case of all the SFRC beams, the longitudinal strain value starts to decline after reaching the initial peak, possibly due to bond failure between the strain gauge and the longitudinal reinforcement under the intense impact load, resulting in inaccurate strain gauge data. Additionally, the longitudinal strain values sharply increase after reaching the initial peak, indicating that the longitudinal reinforcement exceeds the maximum strain value of the strain gauge post-yielding, leading to strain gauge overload. At approximately 7 ms, the longitudinal strain gauges on the SFRC beams fail due to severe tensile damage to the longitudinal reinforcement.

### 4.3. Analysis of Energy Dissipation

**Figure 17:**
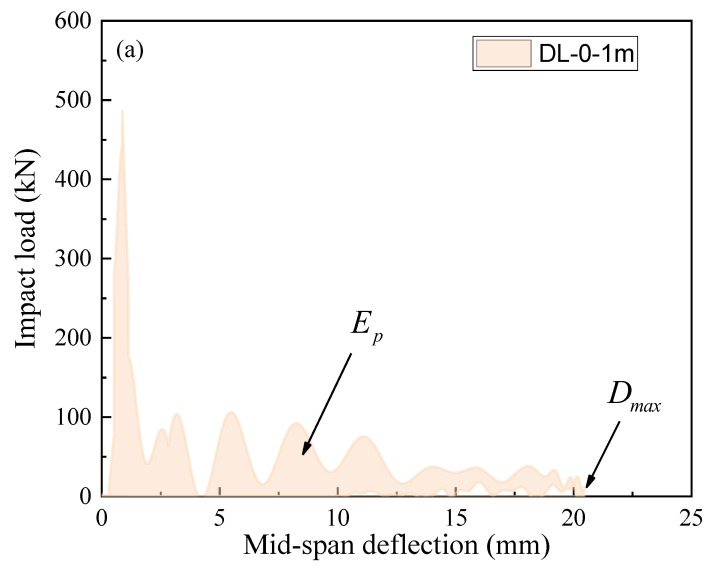

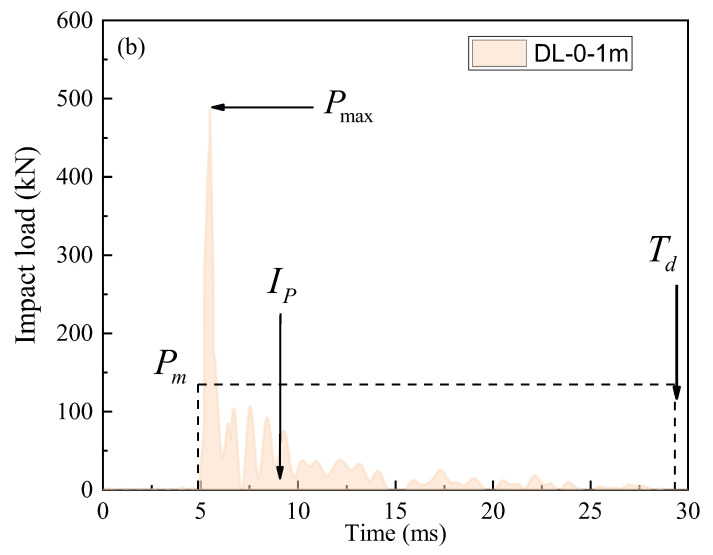
Energy dissipation analysis diagram of SFRC beams in the impact load test. (**a**) Curves of the mid-span deflection and impact load, and (**b**) curves of the time and impact load.

The impact resistance of the SFRC beams is further investigated in this study by integrating the impact load–deflection curves to calculate the total deformation energy, as depicted in Figure 17. The impulse experienced during the impact process is determined by integrating the impact load over the duration of the impact. The average impact load is then computed by dividing the impulse by the duration of the impact. Furthermore, the parameters depicted in Figure 17 can be represented as follows:

(3) $$I_{P}=\int_{0}^{T_{d}}Pdt$$

(4) $$E_{P}=\int_{0}^{D_{\max}}Pd\delta$$

(5) $$P_{m}=\frac{I_{p}}{T_{d}}$$

where $I_{P}$ is the impulse, $E_{P}$ is the energy dissipation, $D_{\max}$ is the peak mid-span deflection, $P_{m}$ is average impact load, and $T_{d}$ is the impact time. 

**Figure 18:**
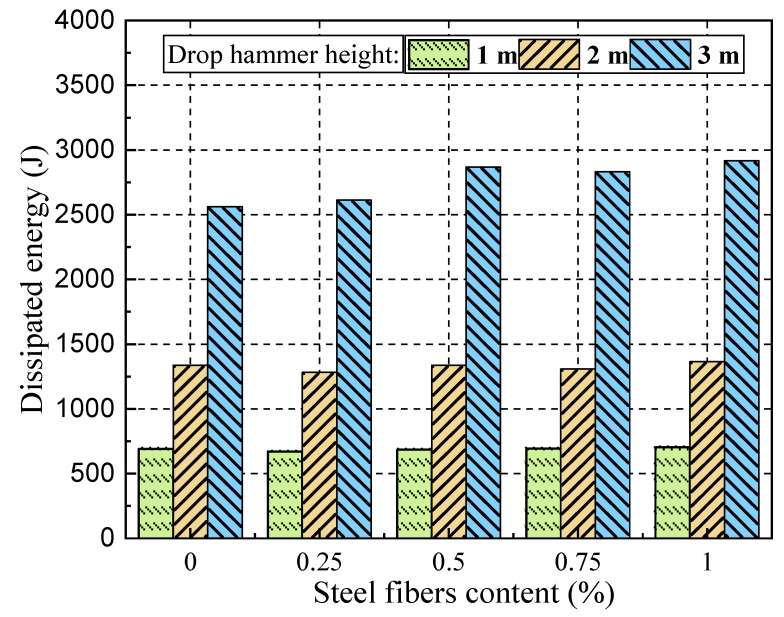
Energy dissipation of SFRC beams with different drop hammer heights in the impact load test.

Table 4 and Table 5 list the parameters of energy dissipation, impulse, average impact force, and peak mid-span deflection for all SFRC beams. With an increase in drop hammer height, the overall energy dissipation of the SFRC beams also increases, as depicted in Figure 18. It is observed that the overall energy dissipation ranges from approximately 600 J to 700 J, 1200 J to 1400 J, and 2500 J to 2900 J with 1 m, 2 m, and 3 m drop hammer heights in the impact load test, respectively. Furthermore, an increase in steel fiber content is associated with a certain degree of increase in overall energy dissipation. For example, the energy dissipation of SFRC beam DL-1-3m is 2916.4 J greater than that of SFRC beam DL-0.25-3m, which is 2612 J. Additionally, Table 5 indicates a noticeable improvement in energy dissipation for SFRC beams with an increase in drop hammer height.

**Figure 19:**
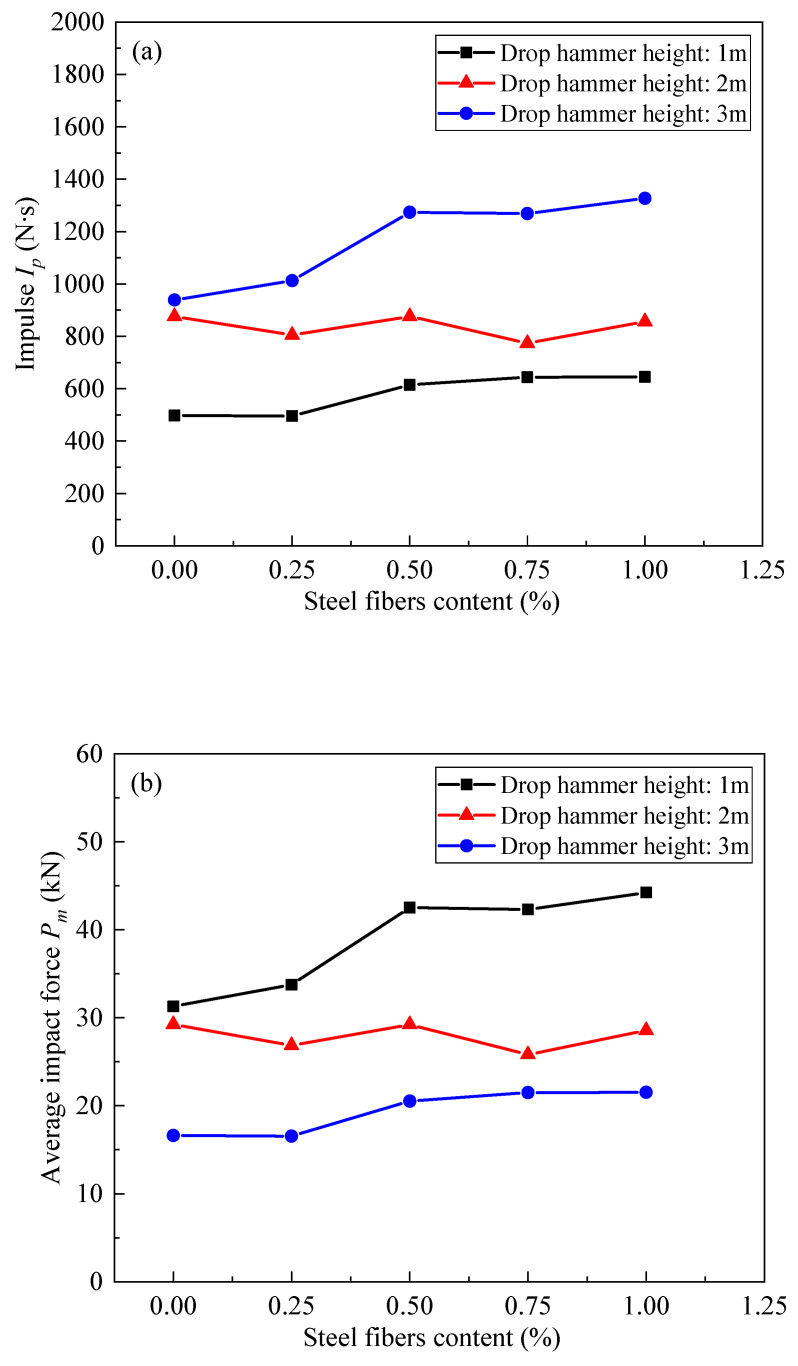
The curves depict the relationship between impulse, average impact force, and steel fiber content for SFRC beams under various drop hammer heights in the impact load test. (**a**) The curves of impulse and steel fibers content. (**b**) The curves of average impact force.

In Figure 19, the correlation between the impulse, average impact force, and steel fiber content in SFRC beams is depicted across different drop hammer heights during the impact load test. As shown in Figure 19a, the impulse $I_{p}$ of the SFRC beams demonstrated an increase with higher steel fiber content during both the 1 m and 3 m drop hammer impact tests, with the effect being more pronounced. However, the results of the 2 m drop hammer impact test exhibited inconsistencies compared to the 1 m and 3 m tests, possibly due to incomplete sensor adherence to the steel bars, leading to data deviation. Additionally, the average impact force $P_{m}$ of the SFRC beams increased with higher steel fiber content across all drop hammer heights, with the effect being more prominent.

## 5. Conclusions

This study examined how the impact load test of corrosion-treated SFRC beams is affected by variations in steel fiber content and drop hammer height. Experiments were conducted with different steel fiber contents (ranging from 0% to 1.0%) and curing periods of 28 days, along with varying drop hammer heights (1 m, 2 m, and 3 m). The failure modes of the SFRC beams were assessed through examining steel fiber content and drop hammer heights, revealing insights into their mechanical behaviors and load resistance capacities. Key strength parameters discussed included peak load, stirrup strain, longitudinal bar strain, impulse, and energy dissipation. The study’s findings are outlined as follows:

1. From the corrosion test results, SFRC beams with added steel fibers exhibit fewer surface rust spot areas compared to those without steel fibers. This is attributed to the bonding of steel fibers with the concrete matrix and their ability to share part of the corrosion current, resulting in fewer severe rust marks on the concrete surface.
2. The impact tests reveal that SFRC beams exhibit concrete crushing at the top without spalling, with flexural and flexure-shear cracks developing at mid-span. Higher steel fiber content correlates with enhanced impact resistance, resulting in fewer cracks. With increased impact loads, a shift towards shear-dominated failure occurs, causing more severe localized damage. High-speed camera analysis confirms that steel fiber inclusion boosts impact resistance by reducing crack spread, enhancing stiffness, modifying failure modes, and mitigating concrete spalling.
3. SFRC beams with different steel fiber contents display varied responses to impact loads at drop heights ranging from 1 m to 3 m. Enhanced steel fiber content correlates with improved overall beam performance, as evidenced by higher peak impact load values and distinctive mid-span deflection characteristics. Moreover, a consistent impulse oscillation zone is noted across various drop heights, indicating an enhanced energy dissipation capacity. Stirrup reinforcement strain gradually increases with drop height, while longitudinal strain response is marginally faster, suggesting strain gauge failure under intense impact loads. In summary, integrating steel fibers into SFRC beams enhances their impact resistance and deformation capacity, thereby improving structural performance under impact loading conditions.
4. The energy dissipation analysis indicates a moderate increase in energy dissipation with higher steel fiber content. Impulse and average impact force generally increase with higher steel fiber content, although some inconsistency is noted in the 2 m drop hammer tests, possibly due to sensor adherence issues. Nonetheless, the trend indicates that higher steel fiber content enhances impulse and average impact force.

## Figures and Tables

**Table 1:** Basic parameters of concrete materials (results of compressive strength tests for concrete cube specimens).

Curing Period (d)	Average Cubic Compressive Strength (MPa)	Axial Compressive Strength (MPa)
14	32.1	24.4
21	34.2	26.0
28	37.6	28.6

**Table 2:** Basic parameters of steel-fiber-reinforced concrete materials (results of compressive strength tests for steel-fiber-reinforced concrete cube specimens).

Type	Steel Fiber Dosage (%)	Curing Period (d)	Average Cubic Compressive Strength (MPa)	Axial Compressive Strength (MPa)
DL-0	0	28	33.00	25.08
DL-0.25	0.25		41.85	31.81
DL-0.50	0.50		42.97	32.66
DL-0.75	0.75		45.63	34.68
DL-1.00	1.0		45.27	34.41

**Table 3:** Experimental program of impact load testing of SFRC beams.

Types	Hammer Mass (kg)	Falling Height (m)	Impact Velocity (m/s)	Corrosion Time (d)	Steel Fibers Content (%)
DL-0-1m	100	1	4.43	48	0
DL-0-2m		2	6.26		0
DL-0-3m		3	7.67		0
DL-0.25-1m		1	4.43		0.25
DL-0.25-2m		2	6.26		0.25
DL-0.25-3m		3	7.67		0.25
DL-0.5-1m		1	4.43		0.5
DL-0.5-2m		2	6.26		0.5
DL-0.5-3m		3	7.67		0.5
DL-0.75-1m		1	4.43		0.75
DL-0.75-2m		2	6.26		0.75
DL-0.75-3m		3	7.67		0.75
DL-1.0-1m		1	4.43		1.0
DL-1.0-2m		2	6.26		1.0
DL-1.0-3m		3	7.67		1.0

**Table 4:** Impact test results under varying conditions of steel fiber content.

Type	Steel Fibers Content (%)	Impact Speed (m/s)	Peak Impact Load (kN)	Peak Mid-Span Deflection (mm)	Residual Mid-Span Deflection(mm)
DL-0-1m	0	4.43	266.92	18.16	12.34
DL-0.25-1m	0.25	4.43	280.62	16.26	9.66
DL-0.5-1m	0.5	4.43	336.47	16.24	9.15
DL-0.75-1m	0.75	4.43	387.53	16.03	8.55
DL-1.0-1m	1.0	4.43	413.86	15.26	6.75
DL-0-2m	0	6.26	541.79	38.45	30.28
DL-0.25-2m	0.25	6.26	556.92	31.92	24.83
DL-0.5-2m	0.5	6.26	610.48	30.65	24.68
DL-0.75-2m	0.75	6.26	665.20	28.07	20.10
DL-1.0-2m	1.0	6.26	720.88	26.87	19.83
DL-0-3m	0	7.67	653.70	55.33	50.69
DL-0.25-3m	0.25	7.67	715.81	45.20	41.22
DL-0.5-3m	0.5	7.67	837.91	41.40	37.29
DL-0.75-3m	0.75	7.67	920.13	38.29	33.81
DL-1.0-3m	1.0	7.67	1051.27	35.63	29.58

**Table 5:** The overall energy dissipation results of SFRC beams in the impact load test with varying factors (drop hammer height and steel fiber content).

Type	Energy Dissipation *E*_p_ (J)	Impulse *I*_p_ (N∙s)	Average Impact Force *P_m_* (kN)
DL-0-1m	691.2	497.7	16.59
DL-0-2m	1336.7	876.5	29.22
DL-0-3m	2562.4	938.5	31.28
DL-0.25-1m	669.4	495.8	16.53
DL-0.25-2m	1281.2	805..2	26.84
DL-0.25-3m	2612.0	1012.5	33.75
DL-0.5-1m	684.7	614.7	20.49
DL-0.5-2m	1335.6	876.2	29.21
DL-0.5-3m	2868.7	1273.6	42.45
DL-0.75-1m	692.4	644.3	21.47
DL-0.75-2m	1307.6	773.8	25.80
DL-0.75-3m	2831.7	1268.8	42.29
DL-1.0-1m	703.1	645.1	21.50
DL-1.0-2m	1365.7	856.3	28.54
DL-1.0-3m	2916.4	1326.8	44.23

## Data Availability

The original contributions presented in the study are included in the article, further inquiries can be directed to the corresponding author.
